# A Randomized, Single-Center Double-Blinded Trial on the Effects of Diltiazem Sustained-Release Capsules in Patients with Coronary Slow Flow Phenomenon at 6-Month Follow-Up

**DOI:** 10.1371/journal.pone.0038851

**Published:** 2012-06-27

**Authors:** Lun Li, Ye Gu, Tao Liu, Yupeng Bai, Lingbo Hou, Zhong Cheng, Liqun Hu, Bo Gao

**Affiliations:** Department of Cardiology, Wuhan Puai Hospital, Huazhong University Of Science and Technology, Wuhan, China; University Medical Center Utrecht, Netherlands

## Abstract

**Objective:**

The aim of this study is to observe the chronic effects of diltiazem release capsules on patients with coronary slow flow (CSF) phenomenon.

**Methods:**

From 2004 to 2009, 80 consecutive patients with chest pain and normal coronary arteries evidenced by coronary angiography and CSF were included in this randomized, double-blind, placebo-controlled trial. CSF patterns were evaluated by the corrected TIMI frame count. Patients were randomly assigned at 1∶1 ratio to diltiazem sustained-release capsules treatment group (Dil, 90 mg twice daily) or placebo control group. Holter, liver and kidney function, treadmill exercise test, coronary angiography and left ventricular angiography were measured at baseline and after 6 months. The incidence of cardiovascular events (re-admission or progress in coronary heart disease, myocardial infarction, malignant arrhythmia or cardiac death) was evaluated during the 6 months follow up.

**Results:**

Thirty-nine patients in control and 40 patients in Dil group completed the 6 months follow-up. There was no medication induced drug withdraw during follow up. Left ventricular ejection fraction was similar between the 2 groups at baseline and during follow up. Heart rate was significantly lower in Dil group than in control group and there was no symptomatic bradycardia and II and III degree atrioventricular conduction block in both groups. Significant improvement was observed in the onset of chest pain, treadmill exercise test and coronary blood flow in Dil group while these parameters remained unchanged in control group at the end of 6 months follow up. The incidence of cardiovascular events was similar between the two groups.

**Conclusion:**

Diltiazem slow-release capsules improved coronary blood flow and alleviated angina in patients with CSF.

**Trial Registration:**

Chinese Clinical Trial Registry ChiCTR-TCC-11001864

## Introduction

In 1972, Tambe [1] first reported coronary slow flow (CSF) phenomenon defining chest pain patients without significant coronary artery lesion but with slowed down coronary blood flow during coronary angiography examination. Mangieri [2] et al. reported that incidence of CSF was around 7% in patients with suspected coronary heart disease. CSF can lead to myocardial ischemia, acute coronary syndrome and acute myocardial infarction [3]. It was suggested that coronary microcirculation obstacle might be the main reason for CSF phenomenon [4], [5], [6]. Previous studies have shown that calcium antagonists could relief microvascular spasm [7], [8] and intravascular application of diltiazem could to attenuate coronary artery spasm in patients with microvascular angina [9], [10]. Effects of chronic oral calcium antagonists on CSF patients remain largely unknown now. This study aimed to observe the chronic effects of oral diltiazem sustained-release capsules in patients with CSF phenomenon. Data of chest pain frequency, 24-hour Holter, treadmill exercise test, coronary angiography and left ventricular angiography at baseline and at the end of 6 months follow up were compared between CSF patients receiving oral diltiazem sustained-release capsules or placebo. Major adverse cardiac events (re-hospitalization; acute coronary syndrome, malignant arrhythmia or cardiac death) during follow-up were also recorded and liver and kidney functions were monitored during the study period.

## Methods

The protocol for this trial and supporting CONSORT checklist are available as supporting information; see Checklist S1 and Protocol S1.

**Figure 1:**
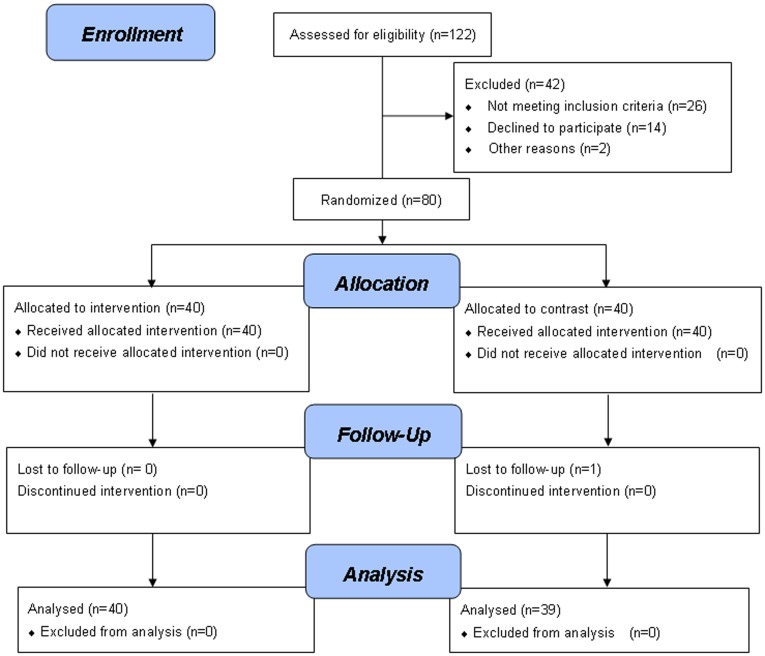
Study design flow diagram.

### Study Patients

Eighty patients with chest pain and diagnosed as CSF using the corrected (corrected TIMI frame count, CTFC) method [11] from 2004 to 2009 in our department were included. Exclusion criteria: 1) A history of myocardial infarction or coronary angioplasty; 2) Cardiomyopathy, valvular disease, hypertensive heart disease, congenital heart disease; 3) Coronary artery dilatation, stenosis (stenosis >40%); 4) Systolic blood pressure <90 mmHg; 5) The resting heart rate <60 times/min, sick sinus syndrome, second or third degree atrioventricular conduction block; 6) NYHA functional class ≥ III; 7) Liver or renal dysfunction (serum aspartate transaminase or alanine transaminase ALT increased by 2-fold, creatinine ≥2 mg/dL); 8) Study drug allergy.

**Table 1:** Patient data.

	Control group(n = 39)	Dil group(n = 40)	P value
Age (years)	50.5±7.8	53.7±7.4	0.07
Gender (male%)	29/39 (74.4%)	30/40(75%)	1
Risk factors			
Hypertension	18/39 (46.2%)	23/40(57.5%)	0.371
Diabetes	13/39 (33.3%)	13/40(32.5%)	1
Hyperlipidemia	26/39 (66.7%)	24/40(60%)	0.642
History	23/39 (59.0%)	24/40(60%)	1
Smoking (>10/day)	19/39 (48.7%)	23/40(57.5%)	0.502
Body Mass Index (kg/m2)	25.0±1.2	25.0±1.1	0.992
ETPR	23/39 (59.0%)	22/40 (55%)	0.821
CSFBV(single/double sticks)			
LAD	23/47 (48.9%)	25/50 (50%)	1
LCX	11/47 (23.4%)	10/50 (20%)	0.806
RCA	13/47 (27.7%)	15/50 (30%)	0.826
CTFC (frames)	55.8±8.9	54.1±8.9	0.273
Ejection fraction (%)	60. 5±5. 6	58.6±5.1	0.136
Chest pain (times/week)	1.9±1.1	1.8±1.2	0.569
Medication			
ACEI/ARB	18/39 (46.2%	23/40 (57.5%)	0.371
Antidiabetic agents	13/39 (33.3%)	12/40 (30%)	0.812
Lipid-lowering agents	25/39 (64.1%)	21/40 (52.5%)	0.364
Beta-adrenoblockers	21/39 (53.8%)	20/40 (50%)	0.823
Nitrates	32/39 (82.1%)	34/40 (85%)	0.770

LAD, Left anterior descending; LCX, Left circumflex artery; RCA, right coronary artery; ETPR, exercise test positive rate; CSFBV, coronary slow flow blood vessel; CTFC, corrected TIMI frame count; ACEI, Angiotensin converting enzyme inhibitor; ARB, Angiotensin-II receptor typy-1 blocker. Unpaired Student’s t test or Mann-Whitney-Test was used to compare differences in means or mean ranks of variables between control group and Dil group.

### Study Protocol

CSF patients were randomly assigned to diltiazem sustained-release capsules treatment group (Dil, 90 mg twice daily, n = 40) or placebo control group (n = 40) at 1∶1 ratio. Drug and placebo were made and numbered 1–80 randomly by Office of Good Clinical Practice of Puai hospital and were identically packaged in capsule form. The code was broken only after the study was completed and investigators remained blind throughout the study, and analysis was conducted by a statistician who had no patient contact. The compliance was determined by pill counts. Pill counts were attempted on all prescribed medications that were to be taken regularly in discrete dosages. Percent adherence was calculated using the following equation: (number of tablets taken/number of tablets that should have been taken) × 100. Both groups stopped using anti-angina drugs (nitrates, beta-adrenoblockers, nifedipine) two weeks before study begin; angina attack could be treated with sublingual isosorbide dinitrate at any time. Patients were followed up for 6 months. Chest pain frequency, 24-hour Holter, treadmill exercise test, coronary angiography and left ventricular angiography were examined at baseline and at the end of 6 months follow up. Major adverse cardiac events (re-hospitalization; acute coronary syndrome, malignant arrhythmia or cardiac death) during follow-up were recorded. Liver and kidney functions were also monitored during the study period. The study was approved by the hospital ethics committee[Appendix S1], written consent[Appendix S2] was obtained from each participating patient.

**Table 2:** Safety Analysis.

	Control group(n = 39)	Dil group(n = 40)
Heart rate (beats/min)		
Baseline	71.5±7.9	74.6±6.8
Follow up	71.0±7.4	69.3±6.8∧ (p<0.001)
Ejection fraction (%)		
Baseline	60.5±5.6	58.6±5.1
Follow up	60.0±4.4	58.5±4.7
Creatinine (mg/dL)		
Baseline	0.70±0.21	0.72±0.18
Follow up	0.71±0.21	0.74±0.18
Alanine transaminase (U/L)		
Baseline	25.5±9.0	24.5±8.1
Follow up	24.8±7.4	25.8±7.1

∧ p<0.05 vs. baseline. Unpaired Student’s t test or Mann-Whitney-Test was used to compare differences in means or mean ranks of variables between control group and Dil group. Paired Student’s t test or Wilcoxon signed-rank test was used to compare the means or mean ranks of the two related samples (baseline vs. Follow up) as indicated. The Bonferroni correction was applied and adjust p-values (p value x 2) were obtained.

**Table 3:** The effects of the treatment.

	Control group(n = 39)	Dil group(n = 40)
CSF (branches)		
Baseline	47	50
Follow up	37 (78.7%)	18 (36.0%)*(p<0.001)
CTFC (frames)		
Baseline	55.8±8.9	54.1±8.9
Follow up	49.4±11.0∧ (p<0.001)	41.2±12.9*(p = 0.002) ∧(p<0.001)
Chest pain (episodes/week)		
Baseline	1.9±1.1	1.8±1.2
Follow up	1.4±1.1	0.7±0.9*(p = 0.004) ∧(p<0.001)
ETPR (%)		
Baseline	23 (59%)	22 (55%)
ST segmentdepression	15	13
Typical anginasymptoms	5	6
Both	3	3
Follow up	19 (48.7%)	10 (25%)∧(p = 0.024)
ST segmentdepression	16	8
Typical anginasymptoms	1	1
Both	2	1

∧ p<0.05 vs. baseline; *p<0.05 vs. control. CSF, coronary slow flow; CTFC, corrected TIMI frame count; ETPR, exercise test positive rate. Unpaired Student’s t test or Mann-Whitney-Test was used to compare differences in means or mean ranks of variables between control group and Dil group. Paired Student’s t test or Wilcoxon signed-rank test was used to compare the means or mean ranks of the two related samples (baseline vs. Follow up) as indicated. The Bonferroni correction was applied and adjust p-values (p value x 2) were obtained.

**Table 4:** MACE during follow up.

	Control group(n = 39)	Dil group(n = 40)	P value
Rehospitalization for chest pain	5	2	0.263
Progress in coronary heart disease	0	0	
Myocardial infarction, malignant arrhythmia or cardiac death	0	0	

Fisher’s exact test (rate comparison) was performed to compare proportions between control group and Dil group.

### Evaluation of Coronary Flow Velocity

Coronary flow velocity was determined by CTFC method. Briefly, PHILIPS CV12 digital subtraction angiography was used for multi-position selective coronary angiography by Judkins method, total frame rate was of 25 frames/s. Left anterior descending coronary artery (LAD) was acquiesced by right anterior oblique 30° value plus foot position 30°, number of frames from the opening of the left anterior descending artery to the apical bifurcation was measured. Right coronary artery (RCA) was acquiesced by left anterior oblique 45°, number of frames from the opening of RCA to left ventricle branch after the first collateral branch bifurcation was measured; Left circumflex artery (LCX) was acquiesced by right anterior oblique 30° plus foot position 30°, number of frames from the opening of left circumflex artery to the distal obtuse marginal branch was measured. According to the Gibson method [11], the number of frames of LAD was divided by 1.55. TIMI-FC <40 was defined as normal flow (normal coronary flow, NCF), ≥40 as slow flow (slow coronary flow, SCF).

### Treadmill Exercise Test

Exercise test was performed at the same time in the morning by the same physician. All patients underwent submaximal exercise treadmill test (ETT) according to the standard Bruce protocol [12]. The protocol continued until one of several endpoints was reached. These included if the patient achieved the target HR [85% of their age-predicted maximal HR  =  (220 − age) × 85%)]. The exercise was terminated in following conditions: developed severe chest pain, fatigue, leg discomfort or dyspnea; developed frequent premature ventricular beats, systolic blood pressure (SBP) >250 mmHg or >10 mmHg SBP drop compared to pretest SBP; or developed any other reasons necessitating termination of exercise. The criteria for ‘positive’ were: ECG showed ST segments of adjacent leads descended horizontally or downslopingly for at least 0.1 mV, and last for more than 2 min, with or without concomitant typical angina symptoms. The criteria for ‘negative’ were: objective load achieved without ST-T changes.

### Sample Size

Calculation of the sample size was determined by using standard methods for binomial data. The effective rate (marked reduction) of CFS at the last day of treatment was the determining factor for sample size calculation. Assuming the estimated effective rate in the control group and Dil group is 20% and 55%, respectively, 35 patients per each group are necessary to detect the statistically significant difference between 2 groups using a 2-sided test at 90% power and α = 0.05.

### Statistics

Continuous data are expressed as mean ± standard deviation. Categorical or dichotomous variables were expressed as percentages. Normality of distribution of all continuous variables was explored by examining skewness, kurtosis, and Q–Q aplots. Unpaired Student’s t test or Mann-Whitney-Test was used to compare differences in means or mean ranks of variables between control group and Dil group. Paired Student’s t test or Wilcoxon signed-rank test was used to compare the means or mean ranks of the two related samples (baseline vs. Follow up) as indicated. Fisheŕs exact test (rate comparison) was performed to compare proportions. The Bonferroni correction was applied when comparing baseline and follow up measures in the control or Dil groups and comparing measures at the baseline and at follow up between control and Dil groups and adjust p-values (p value x 2) were obtained. P value of less than 0.05 was considered to be statistically significant. Statistical analyses were performed using SPSS 14.0 software (SPSS Inc., Chicago, IL, USA).

## Results

A total of 122 patients were screened and 42 patients were excluded due to not meeting inclusion criteria, declined to participate etc. [Figure 1] and the remaining 80 patients were included for the study. All patients (n = 40) in Dil group and 39 patients in control group completed the study and 1 patient in control group was lost to follow up. Baseline clinical and angiographic characteristics were comparable between the two groups (Table 1). The drug compliance was 94.7% in placebo group and 93.8% in Dil group (p>0.05).

Left ventricular ejection fraction, serum creatinine and alanine transaminase remained unchanged during follow up in all patients while average 24 hours heart rate was significantly lower in Dil group than in control group during follow up (Table 2), there was no symptomatic bradycardia, II and III degree AVB at baseline and during follow up. There was no drug withdrawal due to serious side effects. As shown in table 3, coronary flow during follow up returned to normal in 32 out of 50 vessels in Dil group and in only 10 out of 47 vessels in control group (p<0.001). CTFC was improved between baseline and at the end of follow up in both groups and the improvement was more significant in Dil group than in control group. Chest pain frequency (episodes/week) was similar between control and Dil group at baseline and remained unchanged in control group at the end of follow up while it was significantly reduced in Dil group at the end of follow up. Similarly, exercise test positive rate (ETPR) during treadmill exercise test was similar between control and Dil group at baseline and was significantly reduced in Dil group but not in control group after 6 months treatment. There was no deaths, myocardial infarction or need for revascularization procedures. Five patients in control group and 2 patients in Dil group were rehospitalized due to chest pain (Table 4, p>0.05).

## Discussion

Coronary slow flow phenomenon refers to normal coronary arteries with delayed distal perfusion during coronary angiography [13], [14]. The main reason for slow flow phenomenon is considered to microvascular dysfunction [15], [16]. Rim et al [17]. found that coronary slow flow resistance in CFP patients increased significantly compared with the control group. Cell edema, capillary damage and reduced capillary lumen were evidenced in myocardial biopsy of CSF patients by Mangieri et al [18]. and they speculated that these pathological changes might allow a remote increase in vascular resistance resulting in reduced blood flow slow in these patients. Besides the morphological changes, microvascular spasm might also play an important role in the pathogenesis of CSF [19]. Clinical and basic studies have shown calcium antagonists could attenuate microvascular spasm [7], [8], [20]. In line with above findings, our results showed that diltiazem treatment was safe and oral administration of diltiazem sustained-release capsules can improve coronary blood flow and exercise test tolerance, relieve chest pain in patients with CSFP. The most possible mechanism is considered to be the effect on attenuating vascular smooth muscle spasm related to calcium channel blockers. To our knowledge, this is the first clinical report demonstrating the beneficial chronic effects of oral diltiazem release capsules on patients with CSF.

All patients in Dil group completed follow-up, there was no significant difference between the 2 groups at the baseline and the end of follow up on LVEF, serum creatinine and alanine transaminase. There was also no symptomatic bradycardia or second degree and above AVB during follow up. These data suggest that oral administration of diltiazem on patients with CSF is safe.

### Study Limitation

Larger multi-centre studies are warranted to verify the effects of Diltiazem Hydrochloride Sustained-release Capsules in CSF patients obtained from this cohort with small patient number.

### Conclusion

CSF may be a manifestation of microvascular dysfunction. This study found that oral administration of diltiazem sustained-release capsules could improve coronary blood flow and exercise test tolerance, and relieve the chest pain symptoms in patients with the CSF.

## Supporting Information

Protocol S1 **Study protocol.**
(DOC)

Checklist S1 **CONSORT checklist.**
(DOC)

Appendix S1 **Approval about clinical trial for drugs.**
(DOC)

Appendix S2 **Informed consent form.**
(DOC)
