# Supplementary material for: A Randomized, Single-Center Double-Blinded Trial on the Effects of Diltiazem Sustained-Release Capsules in Patients with Coronary Slow Flow Phenomenon at 6-Month Follow-Up
Source: PLoS One. 2012 Jun 27;7(6):e38851. doi: 10.1371/journal.pone.0038851 (PMC3384631; doi:10.1371/journal.pone.0038851)
Supplement: Protocol S1 — Study protocol. (DOC) [file pone.0038851.s001.doc]

**Study protocol**

**1.Objective**: It’s a A randomized, single-center double-blinded trial .The aim of this study is to observe the chronic effects of diltiazem release capsules on patients with coronary slow flow (CSF) phenomenon

**2.Background:** The coronary slow flow(CSF) phenomenon is an angiographic observation characterized by angiographically normal coronary arteries and delayed distal vessel opacification. Mangieri et al. reported that incidence of CSF was around 7% in patients with suspected coronary heart disease. CSF can lead to myocardial ischemia, acute coronary syndrome and acute myocardial infarction . The precise etiopathogenesis of CSF as well as its effective therapy remain unclear.It was suggested that coronary microcirculation obstacle might be the main reason for CSF phenomenon . Previous studies have shown that calcium antagonists could relief microvascular spasm and intravascular application of diltiazem could to attenuate coronary artery spasm in patients with microvascular angina. Effects of chronic oral calcium antagonists on CSF patients remain largely unknown now.

**3.Methods:**

3.1. Ethics:The study was approved by the hospital ethics committee.

3.2. Study patients:

3.2.1. Inclusion criteria: Patients with chest pain and diagnosed as CSF using the corrected (corrected TIMI frame count, CTFC) method from 2004 in Cardiology of Wuhan Puai hospital were included.

3.2.2. Exclusion criteria: 1) A history of myocardial infarction or coronary angioplasty; 2) Cardiomyopathy, valvular disease, hypertensive heart disease, congenital heart disease; 3) Coronary artery dilatation, stenosis (stenosis > 40%); 4) Systolic blood pressure < 90mmHg; 5) The resting heart rate < 60 times / min, sick sinus syndrome, second or third degree atrioventricular conduction block; 6) NYHA functional class ≥ III; 7) Liver or renal dysfunction (serum aspartate transaminase or alanine transaminase ALT increased by 2-fold, creatinine ≥ 2mg/dL); 8) Study drug allergy.

3.2.3. Consent:Written consent was obtained from each participating patient.

3.2.4. Wash-out Period: All participants stopped using anti-angina drugs (nitrates, beta-adrenoblockers, nifedipine) two weeks before study begin; angina attack could be treated with sublingual isosorbide dinitrate at any time.

3.3. Sample size:Eighty patients with CSF should be included .Calculation of the sample size was determined by using standard methods for binomial data. The effective rate (marked reduction) of CFS at the last day of treatment was the determining factor for sample size calculation. Assuming the estimated effective rate in the control group and Dil group is 20% and 55%, respectively, 35 patients per each group are necessary to detect the statistically significant difference between 2 groups using a 2-sided test at 90% power and α = 0.05.

3.4. Divide into groups and Interventions :CSF patients were randomly assigned to diltiazem sustained-release capsules treatment group (Dil, 90mg twice daily, n = 40) or placebo control group (n = 40) at 1:1 ratio.

3.5.Randomisation and Blinding: Drug and placebo were made and numbered 1-80 randomly by Office of Good Clinical Practice of Puai hospital and were identically packaged in capsule form. The code was broken only after the study was completed and investigators remained blind throughout the study, and analysis was conducted by a statistician who had no patient contact.

3.6. Indicator for further observation and Followed up: Patients were followed up for 6 months. Chest pain frequency, 24-hour Holter, treadmill exercise test, coronary angiography and left ventricular angiography were examined at baseline and at the end of 6 months follow up. Major adverse cardiac events (re-hospitalization; acute coronary syndrome, malignant arrhythmia or cardiac death) during follow-up were recorded.

3.6.1. Evaluation of coronary flow velocity:Coronary flow velocity was determined by CTFC method. Briefly, PHILIPS CV12 digital subtraction angiography was used for multi-position selective coronary angiography by Judkins method, total frame rate was of 25 frames/s. Left anterior descending coronary artery (LAD) was acquiesced by right anterior oblique 30° value plus foot position 30°, number of frames from the opening of the left anterior descending artery to the apical bifurcation was measured. Right coronary artery (RCA) was acquiesced by left anterior oblique 45°, number of frames from the opening of RCA to left ventricle branch after the first collateral branch bifurcation was measured; Left circumflex artery (LCX) was acquiesced by right anterior oblique 30° plus foot position 30°, number of frames from the opening of left circumflex artery to the distal obtuse marginal branch was measured. According to the Gibson method [11], the number of frames of LAD was divided by 1.55. TIMI-FC < 40 was defined as normal flow (normal coronary flow, NCF), ≥ 40 as slow flow (slow coronary flow, SCF).

3.6.2. Treadmill exercise test :Exercise test was performed at the same time in the morning by the same physician. All patients underwent submaximal exercise treadmill test (ETT) according to the standard Bruce protocol [12]. The protocol continued until one of several endpoints was reached. These included if the patient achieved the target HR [85% of their age-predicted maximal HR = (220 − age) × 85%)]. The exercise was terminated in following conditions: developed severe chest pain, fatigue, leg discomfort or dyspnea; developed frequent premature ventricular beats, systolic blood pressure (SBP) > 250 mmHg or > 10 mmHg SBP drop compared to pretest SBP; or developed any other reasons necessitating termination of exercise. The criteria for ‘positive’ were: ECG showed ST segments of adjacent leads descended horizontally or downslopingly for at least 0.1 mV, and last for more than 2 min, with or without concomitant typical angina symptoms. The criteria for ‘negative’ were: objective load achieved without ST-T changes.

3.7.Safety: Liver and kidney functions were also monitored during the study period.

3.8. Compliance: Pill counts were attempted on all prescribed medications that were to be taken regularly in discrete dosages. Percent adherence was calculated using the following equation: (number of tablets taken/number of tablets that should have been taken) × 100.

**4.Statistics:** Analysis was conducted by a statistician who had no patient contact. Continuous data are expressed as mean ± standard deviation. Categorical or dichotomous variables were expressed as percentages. Normality of distribution of all continuous variables was explored by examining skewness, kurtosis, and Q–Q aplots. Unpaired Student’s t test or Mann-Whitney-Test was used to compare differences in means or mean ranks of variables between control group and Dil group. Paired Student’s t test or Wilcoxon signed-rank test was used to compare the means or mean ranks of the two related samples (baseline vs. Follow up) as indicated. Fisher´s exact test (rate comparison) was performed to compare proportions. The Bonferroni [correction](app:ds:correction) was applied when comparing baseline and follow up measures in the control or Dil groups and comparing measures at the baseline and at follow up between control and Dil groups and adjust p-values (p value x 2) were obtained. P value of less than 0.05 was considered to be statistically significant. Statistical analyses were performed using SPSS 14.0 software (SPSS Inc., Chicago, IL, USA).

**5.Outcomes:** Major adverse cardiac events (re-hospitalization; acute coronary syndrome, malignant arrhythmia or cardiac death).
