# Supplementary material for: A Randomized, Single-Center Double-Blinded Trial on the Effects of Diltiazem Sustained-Release Capsules in Patients with Coronary Slow Flow Phenomenon at 6-Month Follow-Up
Source: PLoS One. 2012 Jun 27;7(6):e38851. doi: 10.1371/journal.pone.0038851 (PMC3384631; doi:10.1371/journal.pone.0038851)
Supplement: Appendix S1 — Approval about clinical trial for drugs. (DOC) [file pone.0038851.s003.doc]

| Department: Cardiology | Study lead researcher: Pro Ye Gu |
| --- | --- |
| Drug name: Diltiazem Sustained-release Capsule | Form of medication, dosage: [capsule](app:ds:capsule),90mg |
| Whether the drug has been divided into [drug Storage](app:ds:Drug Storage): Yes | |
| Name: Chronic Effects of Diltiazem Sustained-release Capsule in Patients with Coronary Slow  Flow Phenomenon | |
| The sponsor(funding): Puai Hospital,Huazhong University of Science and Technology | |
| Comments of the Review Committee:   1. 1.[Researcher](app:ds:researcher) was qualification requirements. 2. 2. Research programme was [accord with](app:ds:accord with) ethical Principle And Requirements. 3. 3. Informed consent form was [accord with](app:ds:accord with) ethical Principle And Requirements. 4. Conclusion: [Approve](app:ds:approve) this research.   [Institution](app:ds:institution): Puai Hospital,Huazhong University of Science and Technology   1. [chairman of committee](app:ds:chairman of committee)(sign): Guanrong Chen 2. Date:2004.2.20 | |

[**Approval**](app:ds:approval) **about clinical trial for drugs**

Ethics Committee of Puai Hospital,Huazhong University of Science and Technology

ID:2004(003)
