# Supplementary material for: A Randomized, Single-Center Double-Blinded Trial on the Effects of Diltiazem Sustained-Release Capsules in Patients with Coronary Slow Flow Phenomenon at 6-Month Follow-Up
Source: PLoS One. 2012 Jun 27;7(6):e38851. doi: 10.1371/journal.pone.0038851 (PMC3384631; doi:10.1371/journal.pone.0038851)
Supplement: Appendix S2 — Informed consent form. (DOC) [file pone.0038851.s004.doc]

#

**知情同意书**

|  |  |  |  |  |  |  |  |  |  |  |  |  |  |  |  |  |  |
| --- | --- | --- | --- | --- | --- | --- | --- | --- | --- | --- | --- | --- | --- | --- | --- | --- | --- |

姓名: ; 身份证号码:

|  |  |  |
| --- | --- | --- |

出生日期: 年 月 日 研究号:

现在您应该已经确诊存在冠状动脉慢血流现象，并已经经受胸痛等症状的困扰以及可能发生心血管事件。目前对冠状动脉慢血流现象的发生机制及治疗方法还缺乏明确认识，临床及基础研究证明腺苷、硝普钠及钙离子拮抗剂在治疗冠状动脉慢血流现象中起到一定作用，但上述药物均需注射使用，不能保持持久的药效。临床中我们发现口服钙离子拮抗剂地尔硫卓缓释胶囊对改善此类患者症状有一定作用，为进一步证实此药物在冠状动脉慢血流现象的治疗作用，为其他经受同样疾病的患者提供有效安全的治疗方案，我们真诚地邀请您参加本项医学研究。本项医学研究已经得到包括临床和法律专家在内的专门的伦理委员会审批和备案，详细信息参阅《受试者信息》手册。

参加本研究，您将免费获得试验药物半年，以及医生定期严格的随访和评估。请您花一些时间仔细阅读《受试者信息》手册内容，如果您愿意还可以与您的家人商量。是否参加本项研究完全由您自己来决定。如果您不参加，您的医疗服务均不会受到任何影响。

**如果您愿意参加请阅读下列条款并签署您的姓名：**

- 我已阅读《受试者信息》，并理解和接受信息中的内容。我已有机会询问问题，并得到满意的答复。我已有充分的时间考虑是否愿意参加研究。
- 我知道我参加本项研究完全是自愿的，我有权在任何时候退出研究而不影响我的临床治疗和其他权益。
- 我了解研究目的、具体过程与期限、检查内容及预期可能的收益和风险。我已了解参加研究后可能被分配到不同的治疗组别。
- 我知道若发生与研究相关的损害时，我可以获得必要的治疗和补偿。
- 我知道关于我参加本项研究的具体信息，将会以安全、保密的方式保存。
- 我同意在保密的前提下，本项研究的研究人员及研究管理委员会等相关机构人员，可以查看我的医院病历及其他健康记录，以保证研究的正常进行。
- 我知道如果我同意参加本研究，将在今后的6个月内配合研究工作的实施，包括按要求接受随诊检查和服用研究药物。

**我同意并自愿参加地尔硫卓缓释胶囊治疗冠脉慢血流的研究**

受试者签名： 日期

研究者签名： 日期
